# The Synergistic Effects between Sulfobetaine and Hydrophobically Modified Polyacrylamide on Properties Related to Enhanced Oil Recovery

**DOI:** 10.3390/molecules28041787

**Published:** 2023-02-14

**Authors:** Qi Sun, Fu-Tang Hu, Lu Han, Xiu-Yu Zhu, Fan Zhang, Gui-Yang Ma, Lei Zhang, Zhao-Hui Zhou, Lu Zhang

**Affiliations:** 1 Key Laboratory of Photochemical Conversion and Optoelectronic Materials, Technical Institute of Physics and Chemistry, Chinese Academy of Sciences, Beijing 100190, China; 2 University of Chinese Academy of Sciences, Beijing 100049, China; 3 Research Institute of Drilling and Production Technology, PetroChina Qinghai Oilfield Company, Dunhuang 736202, China; 4 State Key Laboratory of Enhanced Oil Recovery (PetroChina Research Institute of Petroleum Exploration & Development), Beijing 100083, China; 5 College of Petroleum Engineering, Liaoning Petrochemical University, Fushun 113001, China

**Keywords:** surfactant, polymer, viscosity, interfacial tension, interfacial dilational modulus, emulsion stability

## Abstract

In order to explore the mechanism responsible for the interactions in the surfactant–polymer composite flooding and broaden the application range of the binary system in heterogeneous oil reservoirs, in this paper, the influences of different surfactants on the viscosity of two polymers with similar molecular weights, partially hydrolyzed polyacrylamide (HPAM) and hydrophobically modified polyacrylamide (HMPAM), were studied at different reservoir environments. In addition, the relationship between the surfactant–polymer synergistic effects and oil displacement efficiency was also investigated. The experimental results show that for HPAM, surfactants mainly act as an electrolyte to reduce its viscosity. For HMPAM, SDBS and TX-100 will form aggregates with the hydrophobic blocks of polymer molecules, reducing the bulk viscosity. However, zwitterionic surfactant aralkyl substituted alkyl sulfobetaine BSB molecules can build “bridges” between different polymer molecules through hydrogen bonding and electrostatic interaction. After forming aggregates with HMPAM molecules, the viscosity will increase. The presence of two polymers all weakened the surfactant oil–water interfacial membrane strength to a certain extent, but had little effect on the interfacial tension. The synergistic effect of the “bridge” between HMPAM and BSB under macroscopic conditions also occurs in the microscopic pores of the core, which has a beneficial effect on improving oil recovery.

## 1. Introduction

Oil is regarded as black gold, the blood of industry, and the necessary driving force for the development of modern industry. The efficient exploitation of oil affects the lifeline of economic social development and is an important guarantee for the prosperity of mankind [1,2,3]. However, with the end of water flooding-dominated secondary oil recovery, two-thirds of the crude oil is still trapped in rock pores in a neuron-like form, so in order to restart and efficiently recover this huge amount of remaining oil, tertiary oil recovery technology came into being [4,5,6,7].

Tertiary oil recovery technology is also known as chemical flooding. After several decades of continuous exploration by scientific researchers, chemical flooding systems have become rich and diverse including alkali flooding, surfactant flooding, polymer flooding, surfactant–polymer binary flooding, alkali–surfactant–polymer ternary flooding, etc. [8,9,10,11,12,13,14]. In order to minimize the damage of the oil displacement agent to the rock pores, meet the actual working conditions of high water content, and the strong heterogeneity of rock pores in reservoirs after water flooding, surfactant–polymer binary composite flooding systems are more and more favored by oil fields [15,16,17,18,19]. 

The polymers used for oil displacement can mainly be divided into two categories: partially hydrolyzed polyacrylamide (HPAM) and hydrophobically modified polyacrylamide (HMPAM). In recent years, functional polymers such as partially branched and partially cross-linked viscoelastic particles (PPG) and viscoelastic microspheres have also appeared to meet the needs of high temperature and high salinity reservoirs [20,21]. The addition of polymers in the chemical flooding system can increase the sweeping efficiency of the oil displacement agent, prevent the occurrence of fingering and channeling, and achieve the effect of “profile control and block” [22,23,24,25]. Furthermore, the capillary bundle “Nc” determines the microscopic displacement efficiency of crude oil retained in the pores of the reservoir rock, so the addition of polymers can effectively increase the viscosity of the displacement fluid and improve the Nc value, thereby achieving the purpose of enhancing oil recovery [26,27,28]. These advantages of polymers have established their indispensable position in oil displacement systems.

Common oil displacement surfactants mainly include anionic surfactants, anionic–nonionic surfactants (extended surfactants), and zwitterionic surfactants, etc. Among them, petroleum sulfonate-based anionic surfactants with a wide range of sources, low prices, and good oil recovery efficiency have become the main choice for conventional oil reservoirs in the past ten years [14,26,29]. However, betaine-based zwitterionic surfactants that exist in the form of “inner salts” in a wide pH range, with low toxicity, good biodegradability, foaming and emulsifying properties, have become a new force targeting high temperature-high salinity reservoirs in recent years [30,31]. The appropriate selection of surfactants for different oil phase properties can reduce the oil-water interfacial tension (IFT) to an ultra-low value (<10^−2^ mN/m) and greatly improve the starting and migration capabilities of the remaining oil in the pores. It is the embodiment of the important role of surfactants in improving oil recovery [32].

The influence of different types of oil-displacing polymers on the interfacial behavior of surfactants has attracted the extensive attention of researchers. Ma and his team investigated the effect of polymers on the ability of anionic and anionic–nonionic surfactants to reduce IFT, and pointed out that the addition of most polymers would change the arrangement of interfacial molecules, resulting in an increase in IFT [33]. Zhu also investigated the effect of HMPAM on the oil–water IFT of anionic–nonionic surfactants, and believed that the increase in IFT was closely related to the number of oxyethylene groups in the surfactant molecules [19]. On the other hand, Li explored the effect of polymers on the interfacial behavior of alkyl sulfobetaines with different structures and found that the addition of HMPAM could form a mixed adsorption membrane with linear betaine on the oil–water interface, resulting in a synergistic effect and a further decrease in the IFT [24]. In addition, Yu et al. considered the influence of the polymer addition on the interfacial dilation parameters of SDS and TX-100 by using the oscillating barriers and IFT relaxation method, respectively. Surfactant molecules will form aggregates similar to mixed micelles with the hydrophobic block of the polymer at the interface. There is a rapid exchange between surfactants and aggregates. The characteristic time of this relaxation process is much shorter than that of diffusion exchange between the bulk phase and interface [34,35,36].

In contrast, significantly fewer studies on the viscosity of the polymer bulk phase by surfactants have been reported. McCormick et al. prepared copolymers of acrylamide (AM) and N-alkylacrylamides, and investigated the effects of nonionic and anionic surfactants on the bulk viscosity of polymers. They found that the addition of surfactant significantly reduced the bulk viscosity of the polymer. The viscosity reduction was attributed to the destruction of the inter-chain structure of the polymer [37]. Bhut et al. explored the effect of the composite surfactant on the bulk viscosity of the polymer in the binary oil flooding system composed by HMPAM and the cationic/nonionic Gemini surfactants. Experimental results show that the hydrophobic block of HMPAM will be surrounded in micelles at a high cationic surfactant concentration; electrostatic repulsions between micelles will reduce the associative strength between polymer molecules, which will also reduce the viscosity of HMPAM [38]. 

In order to make the binary flooding system show a synergistic effect, the addition of a polymer cannot damage the original good interfacial properties of the surfactant to reduce its ability to start and peel off porous crude oil. Polymers may change the original hydrophilic–lipophilic balance of surfactants. In particular, the behavior of hydrophobically modified polymers mixed with surfactants at the oil–water interface to form interfacial aggregates has a great influence on the arrangement of the surfactant molecules. In turn, the presence of surfactants cannot reduce the viscosity of the polymer itself, weakening its effect of expanding the swept volume and adjusting the plugging effect. It should be noted that the hydrophobic tail chain of the surfactant will produce hydrophobic interactions with the functional blocks in the polymer molecule, which has a great influence on the hydrodynamic radius and the overall association strength of the polymer molecule.

In short, most of the works on the effect of surfactants on polymer viscosity are based on conventional surfactants with simple structures [39,40,41]. There have been too few experiments aimed at the effect of betaine-based zwitterionic surfactants with complex molecular structure on the bulk viscosity of different types of oil displacement polymers. In terms of the integrity of the enhanced oil recovery mechanism, both the effect of polymers on the interfacial behavior of surfactants and the influence of surfactants on polymer bulk viscosity under different reservoir conditions have to be considered in a surfactant–polymer binary flooding system, but rarely have studies focused on the latter, which is also pivotal in regulating the properties of crude oil emulsions [42,43,44,45,46].

## 2. Results and Discussion

### 2.1. Effect of Surfactants on the Bulk Viscosity of Polymer Solutions

#### 2.1.1. Concentration Dependence of Viscosity of Flooding Polymers

**Figure 1:**
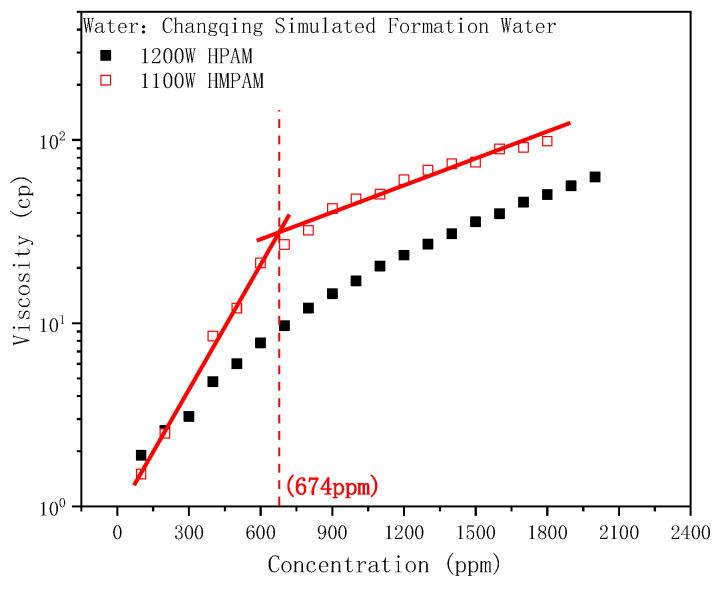
Concentration dependence of the viscosity of HPAM and HMPAM solutions.

The bulk viscosity of HPAM and HMPAM solutions as a function of concentration is shown in Figure 1. The viscosity of both polymers escalated gradually with the increase in concentration, while the viscosity of HMPAM was significantly larger than that of HPAM. Increase in the HPAM viscosity is determined by the size of the polymer molecular chain and the degree of extension in the solution. Aside from the size enhancement effect, HMPAM can also form aggregates through intermolecular hydrophobic interactions to achieve a large viscosity. Increasing the HMPAM concentration strengthens the aggregate structure, thus forming a three-dimensional network structure. The viscosity increased exponentially and the viscosity–concentration curve clearly showed a turning point at the same time, that is, the critical association concentration (CAC) [47,48]. In contrast, HPAM had an approximately linear relationship with viscosity over the entire concentration range without an obvious association phenomenon.

#### 2.1.2. Effect of Anionic Surfactant on Viscosity of Polymer Solutions

**Figure 2:**
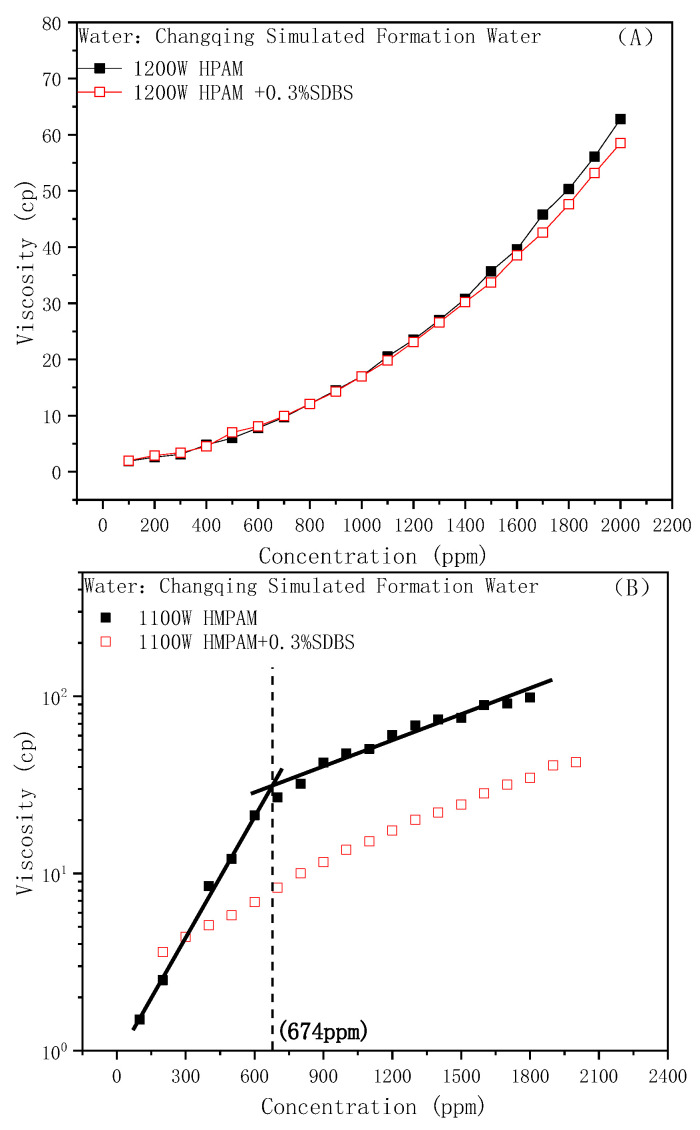
Effect of SDBS on the polymer viscosity: (**A**) HPAM, (**B**) HMPAM.

Under the water conditions of the Changqing Oilfield, the influences of the addition of anionic surfactant SDBS on the viscosity of different types of oil displacement polymers are shown in Figure 2A,B. It can be seen that the sensitivities of the two polymers to SDBS were quite different. The viscosity of HPAM was hardly affected by SDBS at low concentrations and started to decrease slightly with increasing concentration. While the viscosity of HMPAM was greatly reduced, the viscosity–concentration curve was approximately linear in the entire concentration range such as HPAM. There was no CAC, which means that the network structure had been destroyed.

A reduction in viscosity is caused by the “competitive aggregation” behavior of the anionic surfactant: hydrophobic interaction between the hydrophobic group on the surfactant molecule and the hydrophobic block of the hydrophobically modified polymer is stronger than the interaction within HMPAM. SDBS molecules preferentially form aggregates around the hydrophobic block, destroying the original three-dimensional network structure. Furthermore, such aggregates cannot effectively increase the hydrodynamic size and have no structural effect on viscosity.

#### 2.1.3. Effect of Nonionic Surfactant on Viscosity of Polymer Solutions

**Figure 3:**
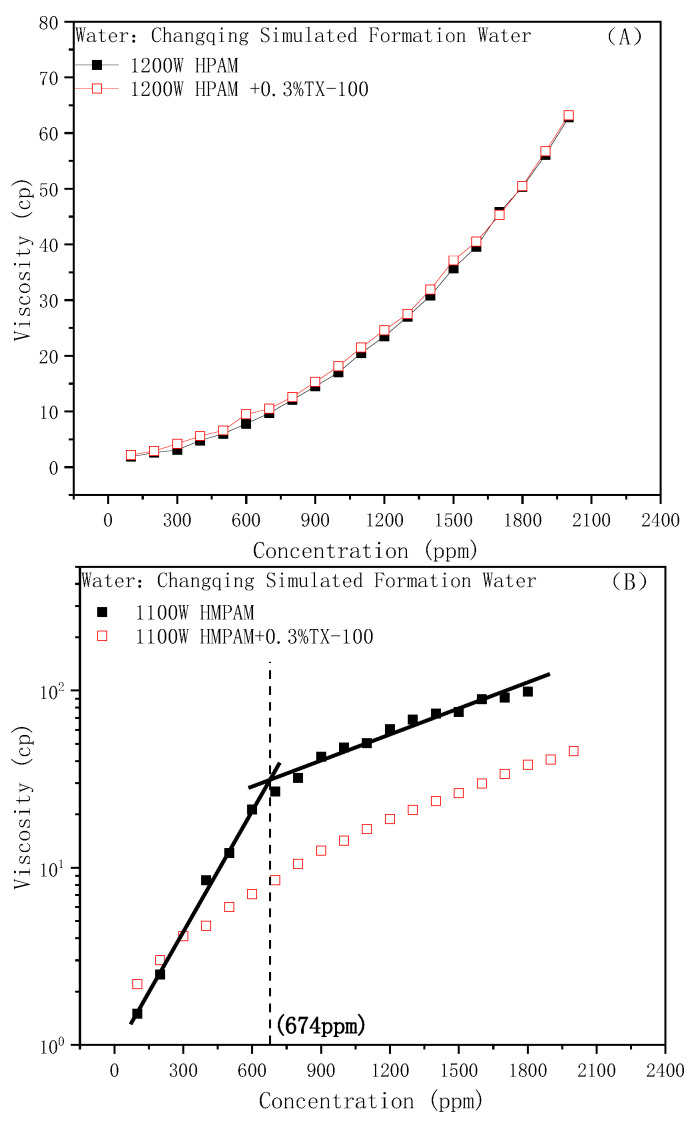
Effect of TX-100 on the polymer viscosity: (**A**) HPAM, (**B**) HMPAM.

Similarly, the effect of nonionic surfactant TX-100 on the viscosity of the HPAM and HMPAM solutions are shown in Figure 3A,B, respectively. Similar to anionic surfactants, the addition of nonionic surfactants will also cause the destruction of the association and strength of the hydrophobically modified polymer, thus the CAC point will no longer appear. Interestingly, the addition of TX-100 did not affect the viscosity of HPAM at all, even at high polymer concentrations compared with SDBS. This could be attributed to the difference of whether the surfactant hydrophilic group is charged or not. It is well-known that the viscosity-increasing effect of HPAM comes from its large hydrodynamic radius. An anionic surfactant can ionize in an aqueous solution, increase the ionic strength of the water phase, and cause the electrostatic repulsion in polymer chains to be shielded by the salt effect. The hydrodynamic size will be compressed, and the bulk viscosity will reduce [49]. In other words, the ionic surfactant mainly acts as an electrolyte to affect the viscosity of HPAM.

#### 2.1.4. Effect of the Zwitterionic Surfactant BSB on the Viscosity of Polymer Solutions

**Figure 4:**
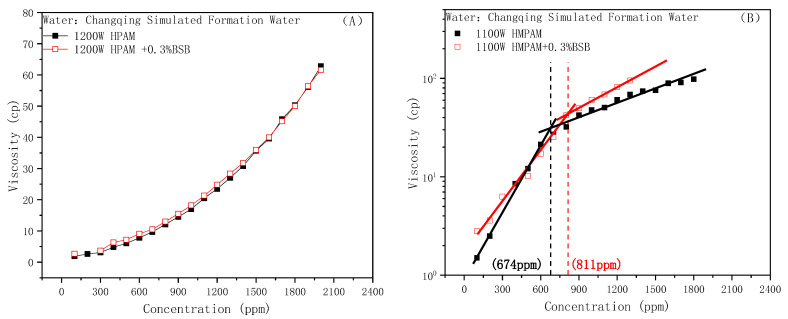
Effect of BSB on the polymer viscosity: (**A**) HPAM, (**B**) HMPAM.

The BSB molecule exists in the form of an “inner salt” in a wide pH range, and the mechanism that affects the viscosity of HPAM is the same as nonionic surfactants, as shown in Figure 4A. However, the experimental results of the effect of BSB on the viscosity of HMPAM showed a completely different trend from those of SDBS and TX-100. As shown in Figure 4B, the addition of BSB did not cause the disappearance of the HMPAM association phenomenon. The CAC value of the system increased from 674 ppm to 811 ppm. Furthermore, the slope of the fitted line after CAC was significantly higher than that without the addition of the surfactant, which indicates a stronger viscosity-increasing effect. That is to say, BSB increases the concentration requirement for 3D network formation; furthermore, once the associative structure is formed, the strength will be greatly improved.

**Figure 5:**
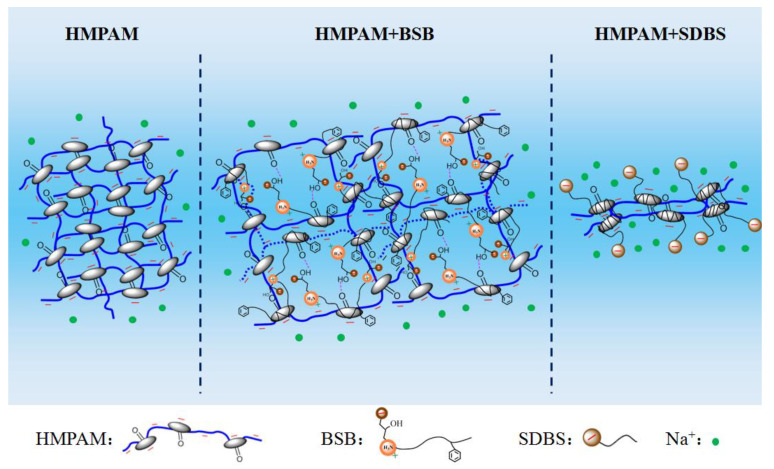
Schematic diagram of the mechanism for the effect of BSB and SDBS on the viscosity of the HMPAM solution.

The phenomenon of viscosity increase is closely related to the special molecular structure of BSB. A slight increase in the CAC value means that the behavior of “competitive association” also exists, the difference being that there is no electrostatic repulsion between BSB molecules. Once aggregates, through hydrophobic interactions with the hydrophobic blocks of HMPAM, form, the hydroxyl group in the hydrophobic group can form a hydrogen bond with the amide group of the polymer, so the positively charged center in the hydrophilic group can generate electrostatic attraction with the negatively charged chain link of the polymer. Coupled with the intermolecular interaction of the BSB molecules itself, which builds a “bridge” between different HMPAM molecules, it maintains and enhances the associative strength of the three-dimensional network structure. As a result, the polymer has a better tackifying effect. The corresponding mechanism is shown in Figure 5.

#### 2.1.5. Effect of Zwitterionic Surfactant BSB on the Polymer Viscosity under Different Water Conditions

Table 1 shows the ionic formulation of the simulated formation water of the Xinjiang and Dagang Oilfields in China. Compared with the Changqing reservoir conditions, the salinity of the two formation waters increased in turn. At the same time, the simulated formation water in Xinjiang had a certain weak alkalinity due to the existence of carbonate.

**Figure 6:**
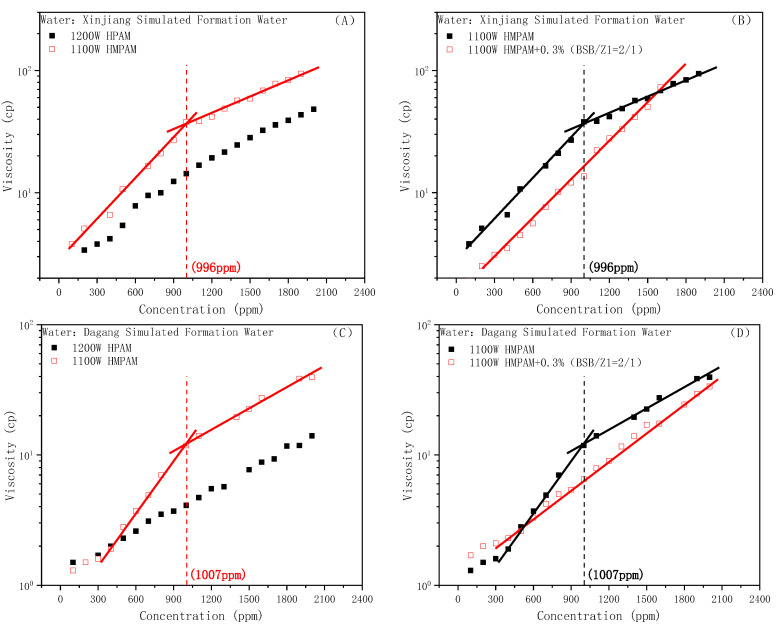
Viscosity of the HPAM and HMPAM solutions under the Xingjiang (weak alkali) condition (**A**). Effect of BSB on the viscosity of HMPAM under the Xingjiang condition (**B**). Viscosity of the HPAM and HMPAM solutions under the Dagang (high salinity) condition (**C**). Effect of BSB on the viscosity of HMPAM under the Dagang condition (**D**).

The viscosity–concentration curves of the HPAM and HMPAM solutions under different water conditions are shown in Figure 6A,C, respectively. With the increase in the ionic strength, the CAC value of HMPAM also gradually increased, showing a certain positive correlation, which is consistent with the findings of Kang et al. [49]. Additionally, the viscosity-increasing behavior of the BSB molecule on hydrophobic polymers was verified under different water conditions. The experimental results are shown in Figure 6B,D, in which Z1 is a polyether nonionic surfactant used to increase the solubility of BSB [28]. It can be seen that the viscosity-increasing effect of BSB on HMPAM still exists, the mixed system with a high polymer concentration shows that it is infinitely close to or has exceeded the viscosity of the polymer alone, and also clearly showed a continuing upward trend. The only difference from the Changqing system is that the CAC of the mixed system did not appear in the whole polymer concentration range. It could be that the presence of the nonionic surfactant increases the difficulty of associative structure formation and the CAC value is delayed toward higher polymer concentrations.

### 2.2. Effect of Polymers on the Oil–Water Interface Behavior of Surfactants

#### 2.2.1. Effect on Oil–Water IFT 

The level of oil–water IFT is a direct representation of the number of surfactant molecules adsorbed on the interface. In addition, the presence of natural active components in crude oil, especially under alkaline water conditions, will have a certain effect on the tightness of the interfacial membrane. Therefore, the research on the effect of different types of oil-displacing polymers on the ability of surfactants to reduce the oil–water IFT becomes very complicated.

**Figure 7:**
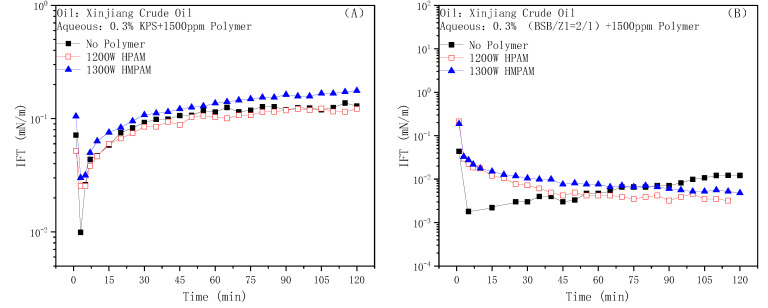
Effect of HPAM and HMPAM on the IFTs of the KPS (**A**) and BSB (**B**) solutions against Xinjing crude oil.

Figure 7A shows the dynamic IFT data of the anionic surfactant petroleum sulfonate KPS against Xinjiang crude oil. The curve gradually rose after a minimum value appeared rapidly, and finally reached a plateau. The addition of the two polymers had little effect on the steady state value of the IFT, but the “V” shape of the dynamic IFT curve became insignificant. According to previous work [27], the IFT between the petroleum sulfonates and n-decane is about 1 mN/m. KPS does not show strong interfacial activity itself, and the existence of intermolecular electrostatic repulsion prevents it from forming a tight film at the oil–water interface, while acidic components in crude oil can synergize with it through mixed adsorption to achieve the effect of greatly reducing the IFT. As for the first decrease and then increase of the dynamic IFT, it was the result of the saponification of petroleum acid on the interface escaping to the water phase [28]. HPAM does not have interfacial activity and mainly regulates the adsorption amount of the KPS molecules or active components on the interface by affecting mass transfer. On the other hand, HMPAM has a certain interfacial activity. The competitive adsorption of HMPAM molecules and the formation of mixed aggregates by HMPAM and the surfactant molecules at the interface slightly improve the steady-state value of IFT [28]. 

Meanwhile, the dynamic IFT between BSB and Xinjiang crude oil is shown in Figure 7B. The hydrophilic and hydrophobic groups of the BSB molecule occupy equal spaces in the oil and water phases, respectively, and have high interfacial activity, which can reduce the decane–water IFT to 0.1 mN/m [50]. More importantly, as BSB molecules can form an extremely close mixed adsorption membrane with small molecular acid components in crude oil at the oil–water interface, the adsorption vacancies at this time on the oil–water interface are extremely small compared to the KPS system. The low interfacial activity of HPAM and the large molecular size of HMPAM both severely restrict their ability to destroy the membrane, so the IFT can still be as low as before after the addition of two polymers. Furthermore, the enrichment of the BSB molecule at the oil–water interface has a significantly higher priority than that of the polymer molecules, and the mass transfer difficulty caused by bulk viscosity reduces the desorption rate of active components at the interface, which is an advantage for keeping the IFT ultra-low.

#### 2.2.2. Effect on Interfacial Dilational Modulus

As we all know, the value of the oil–water IFT determines the difficulty of forming a crude oil emulsion, but the stability of the emulsion is mainly controlled by the strength of the interface membrane. There are a few ways to characterize the strength of the oil–water interface film, among which interface dilation rheology has been proven to be an important method [51]. Sun et al. believed that the process of emulsion rupture can be described as the formation of “holes” on the oil–water interface, while the difficulty of deformation in the expansion region is an intuitive reflection of the interface dilation modulus, too small a modulus is detrimental for maintaining the stability of the emulsion [17].

**Figure 8:**
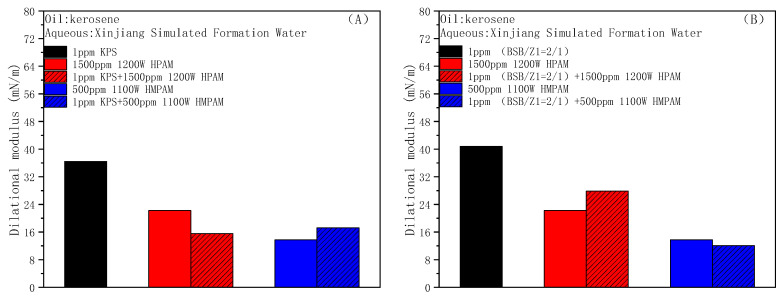
Effects of HPAM and HMPAM on the interfacial dilational modulus of KPS (**A**) and BSB (**B**).

Figure 8A,B shows the effect of two oil displacement polymers, HPAM and HMPAM, on the dilational modulus of the anionic surfactant KPS and zwitterionic surfactant BSB in turn. In the process of interface expansion and compression, the adjustment in the BSB ion head orientation will produce energy storage, and the modulus is slightly higher [52]. The addition of polymers both reduce the dilational modulus of different types of surfactant systems, and the reduction caused by HMPAM is larger because a hydrophobic polymer with a three-dimensional network structure is easier to combine with surfactant molecules through hydrophobic interaction, forming aggregates similar to mixed micelles at and near the oil–water interface. There is a fast exchange between the surfactant molecules and aggregates, and the characteristic time of this relaxation process is much shorter than the diffusion of molecules between the bulk phase and the interface. When there are increases in the interfacial area, the surfactant molecules in the mixed micelles will be rapidly released, and the IFT gradient can be quickly eliminated in situ within the interfacial layer, thereby greatly reducing the interfacial dilational modulus [53].

### 2.3. Effect of Polymers on the Properties of Emulsion

**Figure 9:**
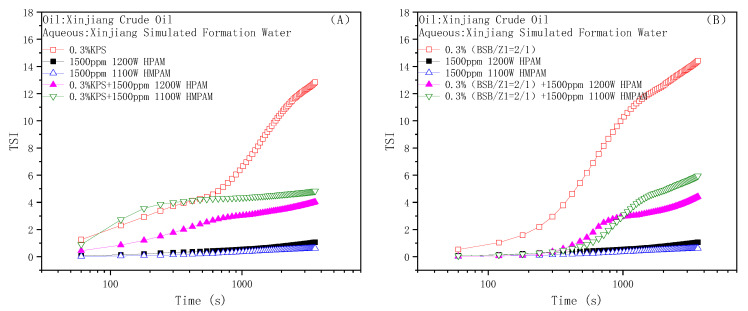
The evaluation of emulsion stability by dynamic light scattering: Binary flooding system with KPS (**A**) and BSB (**B**) as surfactant.

**Figure 10:**
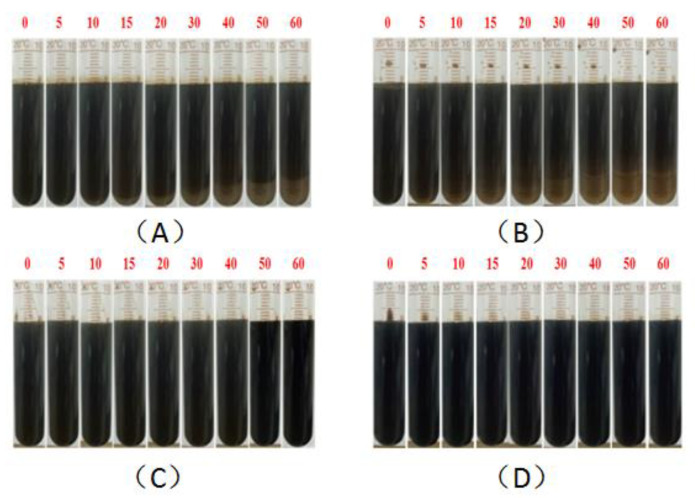
Evaluation of emulsion stability by the bottle test: 0.3% KPS (**A**), 0.3% (BSB/Z1 = 2/1) (**B**), 1500 ppm 1200 W HPAM (**C**), 1500 ppm 1100 W HMPAM (**D**).

**Figure 11:**
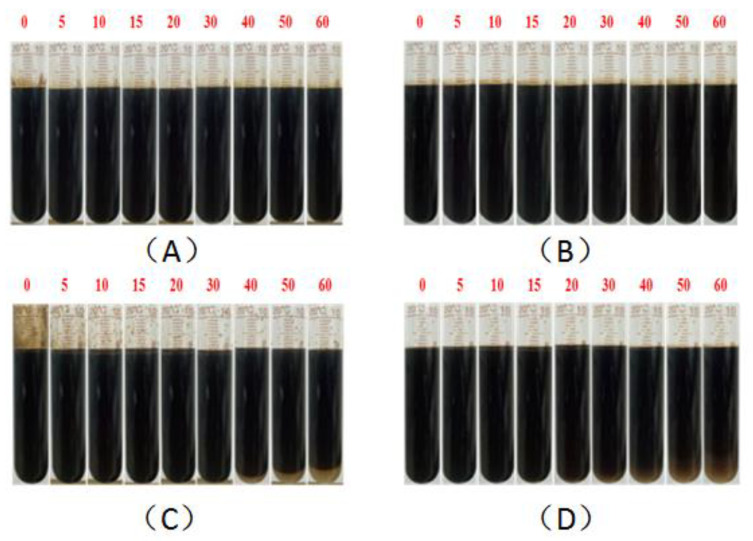
Evaluation of emulsion properties of binary systems by the bottle test: 0.3% KPS + 1500 ppm 1200 W HPAM (**A**), 0.3% KPS + 1500 ppm 1100 W HMPAM (**B**), 0.3% (BSB/Z1) + 1500 ppm 1200 W HPAM (**C**), 0.3% (BSB/Z1) + 1500 ppm 1100 W HMPAM (**D**).

Stability is an important property and evaluation index of a crude oil emulsion, and the starting, sweeping, and migration behaviors of the emulsion in the reservoir rock channels all require certain stability as support [54]. As summarized in the above studies about the interaction of surfactants and polymers in the binary composite flooding system, especially the special property of BSB in increasing the viscosity of the hydrophobic polymer, the emulsion stability was evaluated by the bottle test and dynamic light scattering method. The oil–water mass ratio before emulsification was 1:1. The experimental results are shown in Figure 9, Figure 10 and Figure 11, respectively.

The results clearly show, whether KPS or BSB, that the stability of the emulsion formed by surfactants alone was not very good, indicating that the strength of the interface membrane is not high enough to support the original dispersion state, so the droplets coalesce and gradually separate phases over time. However, emulsions formed by the individual polymers HPAM or HMPAM with Xinjiang crude oil have good stability, which can be attributed to the higher bulk viscosity. The combined effect of the polymer “reducing the interfacial membrane strength” and “increasing the bulk viscosity” leads to the final stability of the binary composite system between the two states. Excitingly, the TSI value of the BSB-containing binary composite system remained almost zero in the early stage, which is an intuitive manifestation of the increase in BSB on the bulk viscosity of HMPAM.

### 2.4. Comparison of Different System Flooding Effects

**Figure 12:**
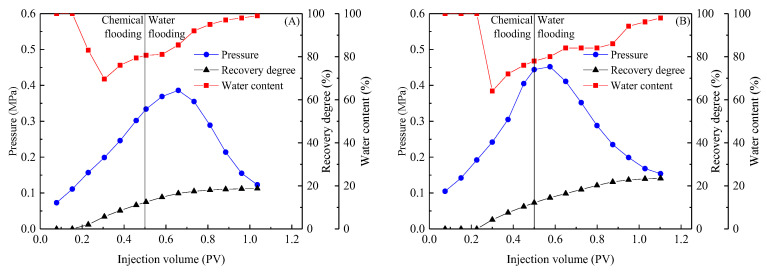
Oil displacement tests with isoconcentration chemical systems: 0.3% KPS + 1500 ppm 1100 W HMPAM (**A**), 0.3% (BSB/Z1) + 1500 ppm 1100 W HMPAM (**B**).

Equal concentrations of the KPS + HMPAM and (BSB/Z1) + HMPAM systems were used to conduct flooding experiments in conglomerate cores to reflect the combined effect of the differences between the two systems on the oil displacement process. The results of the displacement tests are shown in Figure 12.

The pressure changes during flooding can be used to analyze the effect of the different properties of composite systems on the fluids flowing in the core. The injection pressure of the KPS + HMPAM system was consistently lower than that of the (BSB/Z1) + HMPAM system during chemical flooding and subsequent water flooding. This ranking correlates well with the solution viscosity and emulsion stability results as assessed by TSI. One reason for this correlation is that emulsions of different stability, controlled by solution viscosity, can be formed when systems are sufficiently stirred with the crude oil in the porous medium. The emulsion stability of the (BSB/Z1) + HMPAM system was higher than that of the KPS + HMPAM system. These emulsion droplets, which maintain the original scale for a longer time, can more effectively block the microscopic macropores and activate the remaining oil in other pores. Additionally, the higher viscosity of the (BSB/Z1) + HMPAM system facilitates control of the flow ratio, which is another reason for this correlation. The more stable emulsion and higher viscosity of the (BSB/Z1) + HMPAM system further expands the sweep volume, resulting in a higher crude oil recovery of this system than that of the KPS + HMPAM system, which is also reflected in Figure 12. Based on these results, it can be demonstrated that BSB molecules can build “bridges” between different polymer molecules through hydrogen bonding, and electrostatic interaction, previously analyzed under macroscopic conditions, also occurs in the microscopic pores.

## 3. Experimental Section

### 3.1. Materials

**Figure 13:**
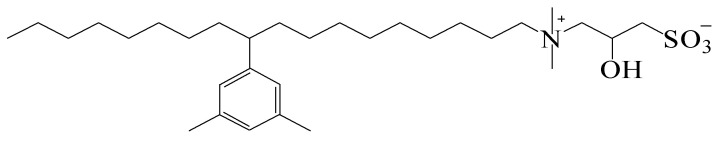
The structure of aralkyl substituted alkyl sulfobetaine (BSB).

The anionic surfactant sodium dodecyl benzene sulfonate (SDBS) and nonionic surfactant TX-100 came from Shanghai Macklin Reagent Company, China. The anionic surfactant petroleum sulfonate (KPS) and the zwitterionic surfactant aralkyl substituted alkyl sulfobetaine (BSB) were provided by the China Petroleum Exploration and Development Research Institute and the structural formula shown in Figure 13. The cmc of BSB and KPS were 1.4 × 10^−6^ mol/L and 6.6 × 10^−3^ mol/L, respectively, at room temperature (25 °C) in distilled water. The critical micelle concentration of BSB was not affected by salinity, while KPS decreased slightly with the increase in salinity. The oil displacement polymer partially hydrolyzed polyacrylamide (HPAM) came from the Daqing Oilfield, China, with a molecular weight of 12 million and a degree of hydrolysis of about 20%. Two kinds of hydrophobically modified polyacrylamides (HMPAMs), which had approximately the same molecular weight (11 million and 13 million), degree of hydrolysis as HPAM, and the content of hydrophobic monomer of 1.5%, were obtained from Beijing Hengju Chemical Group Co. Ltd. (Beijing, China).

The n_min_ values of BSB and KPS obtained by the n-alkane interfacial tension scanning, which can evaluate the hydrophilic–lipophilic balance of surfactants, were 12 and 11, respectively. The larger the n_min_ value, the stronger the oil solubility of the surfactant. Therefore, they have a certain solubility in both oil and water phases. For an ordinary hydrolyzed polymer, due to its good water solubility, there may be a synergistic effect between oil-soluble surfactants and water-soluble polymers to reduce interfacial tension; for hydrophobically modified functional polymers, the hydrophobic groups of the surfactant interact with the hydrophobic blocks in the polymer to form the interior of the “mixed micelle”, and the ion head is exposed to the outside to form a package, which is helpful to promote its dissolution in an aqueous solution.

Three reservoir environments were investigated in this paper. The reservoir temperatures of the Changqing, Xinjiang, and Daqing Oilfields were 50 ± 0.5 °C, 42 ± 0.5 °C, 70 ± 0.5 °C, respectively corresponding, to the simulated formation water formulations shown in Table 1. In addition, the four-component contents and acidic value of Xinjiang crude oil are shown in Table 2. 

### 3.2. Apparatus and Methods

The viscosity test was completed by the rotational rheometer HAAKE MARS II (Thermo Fisher, Germany), with a rotor type of C60/1°Ti, and the speed fixed at 7.34 1/s. Dynamic oil–water IFT was measured by the spanning drop method with a TX-500C meter from Beijing Shengwei Industrial Technology Co. Ltd., and the steady state value of the IFT was considered to be reached when the change in IFT within 30 min was less than 1% [3]. The interfacial dilational modulus [1,55], defined as the change in IFT with the interface area, was measured by the oscillating drop method, using an optical dilational rheometer LSA100OEDM obtained from Beijing Eastern-Dataphy Instrument Co. Ltd. Emulsion stability was characterized by a TURBISCAN Lab stability analyzer based on the principle of dynamic light scattering from Formulaction Company, France. “TSI” is called the stability index and calculated as in Equation (1):

(1) $$\mathrm{TSI}=\sqrt{\frac{\sum_{i=1}^{n}{\left(x_{i}-x_{BS}\right)}^{2}}{n-1}}$$

where *x_i_* is the average value of the backscattered light intensity per scan; *x_BS_* is the average value of *x_i_*; and *n* is the number of scans. The smaller the TSI value, the better the stability of the emulsion [56,57,58,59].

### 3.3. Core Flooding Experiments

At a room temperature of 25 °C, a gas permeability of 300 × 10^−3^ μm^2^ conglomerate core was evacuated for 4 h and saturated with Xinjiang simulated formation water, and the pore volume and porosity were calculated. The length, width, and height of the core were 30.0 × 4.5 × 4.5 cm. The core was then heated at a constant temperature of 42 °C for 12 h. Xinjiang crude oil was used to displace water and the original oil saturation was calculated. Water flooding, chemical slug injection, and subsequent water flooding were performed separately in sequence. Water flooding and subsequent water flooding were stopped when the water content at the outlet reached 98%. Dynamic pressure and oil recovery were recorded during displacements. The injection rate was constant at 0.35 mL/min during the experiments. The composition of the chemical slugs used for the flooding and core data before chemical flooding are shown in Table 3. In polymer slugs, the viscosity was 30 mPa·s.

## 4. Conclusions

In this paper, the effect of different types of surfactants on the bulk viscosity of oil-displacing polymers was taken as the starting point, and the regulation of polymers on the oil–water interface behavior of surfactants was also investigated at the same time. The interactions in the polymer–surfactant binary systems were thoroughly explored and some complete and reliable interaction mechanisms were obtained. Additionally, this was eventually verified by evaluating the emulsion stability and conducting core flooding experiments. This provides a powerful reference for the selection of oil displacement agent formulations in reservoirs with high water content and strong heterogeneity. Some basic conclusions are as follows:

1. Surfactants mainly act as electrolytes to affect the viscosity of the HPAM solution. Uncharged nonionic surfactant TX-100 and electrically neutral zwitterionic surfactant BSB hardly changed the bulk viscosity of HPAM, and the anionic surfactant SDBS caused the viscosity to decrease. The influence of surfactants on the HMPAM bulk viscosity was mainly achieved through hydrophobic interactions, the difference being that the small molecule conventional surfactants SDBS and TX-100 will lead to a disintegration in the HMPAM association structure, but BSB showed a stronger tackifying effect after CAC.

2. HPAM with no interface activity mainly reduced the diffusion and exchange rate of surfactant molecules near the interface by the viscosity effect, and had little effect on the steady-state value of the IFT. However, the ability of HMPAM to regulate the IFT was closely related to the interfacial activity of the surfactants and the tightness of the interfacial membrane. The IFT of a tight membrane was less affected. Otherwise, the steady-state value of the IFT will increase. Furthermore, the presence of HMPAM greatly reduced interfacial dilational modulus of the surfactants.

3. The binary system composed of aromatic alkyl substituted sulfobetaine BSB and the polymer HMPAM had a good synergistic effect. The emulsion formed by the mixed system and crude oil had excellent stability in the short-term, and had good water separation effect when reaching the steady-state, which is beneficial to improving the recovery of crude oil and reducing the difficulty of the emulsion post-treatment. At the same time, the macroscopic thickening effect produced by BSB “bridging” HMPAM was verified in the core displacement experiments, showing the ideal oil displacement effect.

## Figures and Tables

**Table 1:** Changqing simulated formation water formula (mg/L).

	Na^+^ + K^+^	Ca^2+^	Mg^2+^	Cl^−^	SO_4_^2−^	HCO_3_^−^	CO_3_^2−^	TDS
Changqing Oilfield	1504	20	10	479	2318	109	0	4438
Xinjiang Oilfield	3277	24	18	2594	723	3241	117	9993
Dagang Oilfield	10,943	443	65	17,325	284	527	0	29,587

**Table 2:** Four-component contents and acidic value of Xinjiang crude oil.

	Parameter	Resins (wt%)	Asphaltenes (wt%)	Saturates (wt%)	Aromatics (wt%)	Acidic value (mg KOH/g)
Crude Oil						
Xinjiang		8.62	5.50	75.38	10.50	0.2

**Table 3:** The composition of the chemical slugs.

Category of Chemical Flooding	Core Porosity (%)	Initial Crude Oil Saturation (%)	First Water Flooding Recovery (%)	Slug Composition
KPS + HMPAM flooding	18.09	59.24	29.34	0.3 PV (KPS + HMPAM) + 0.2 PV (HMPAM)
(BSB/Z1) + HMPAM flooding	18.38	58.55	30.09	0.3 PV ((BSB/Z1) + HMPAM) + 0.2 PV (HMPAM)

## Data Availability

Not applicable.
